# A review on ultrasound-enhanced heat transfer

**DOI:** 10.1016/j.ultsonch.2025.107570

**Published:** 2025-09-13

**Authors:** Chong Li, Zufen Luo, Yuchen Shao, Yuqi Qian, Siliang Du, Quanquan Yang, Zhong Chen, Hao Chen, Yi Zha, Xiande Fang

**Affiliations:** a Jiangsu Unmanned Aerial Vehicle Research Institute, Faculty of Mechanical and Material Engineering, Huaiyin Institute of Technology, 1 Meicheng Rd., Huaian 223003, China; b China Aviation Life-support Research Institute, 104 Xinhua Rd., Xiangyang 441000, China; c Key Laboratory of Aircraft Environment Control and Life Support, MIIT, Nanjing University of Aeronautics and Astronautics, 29 Yudao St., Nanjing 210016, China

**Keywords:** Ultrasound-enhanced heat transfer, Acoustic cavitation, Acoustic streaming, Influencing parameters, Synergistic effects

## Abstract

This review comprehensively examines recent advances in ultrasound-enhanced heat transfer, a promising active cooling technology for high-heat-flux electronic devices. It systematically analyzes the fundamental mechanisms: thermal effect, acoustic cavitation, acoustic streaming, acoustic fountain and atomization. Among them, acoustic cavitation and acoustic streaming are identified as the two primary mechanisms for enhancing heat transfer. In addition, the review discusses their roles in improving heat transfer in single-phase flow, pool boiling, forced convective boiling, and heat exchanger. Key influencing parameters, such as ultrasonic frequency, power, transducer configuration, flow rate, heat flux, and subcooling are critically evaluated. The synergistic effects of combining ultrasound with nanofluids, channel structure, and other active methods are also highlighted. Numerical modeling approaches, including bubble dynamics and multiphysics simulations, are reviewed for their potential in exploring underlying mechanisms and optimizing system performance. Finally, current challenges and future research directions are outlined, with a focus on multiscale coupling, energy efficiency, and adaptability under extreme conditions.

## 1 Introduction

With the continuous advancement of chip integration, electronic devices are becoming increasingly miniaturized while their heat flux rises significantly. Studies have shown that when device temperatures reach 70-80°C, every 1°C increase reduces their reliability by 5 % [1]. Thermal dissipation performance has become a critical factor affecting the device reliability and operational efficiency of electronic devices, particularly in high-tech fields such as defense and aerospace.

**Fig. 1:**
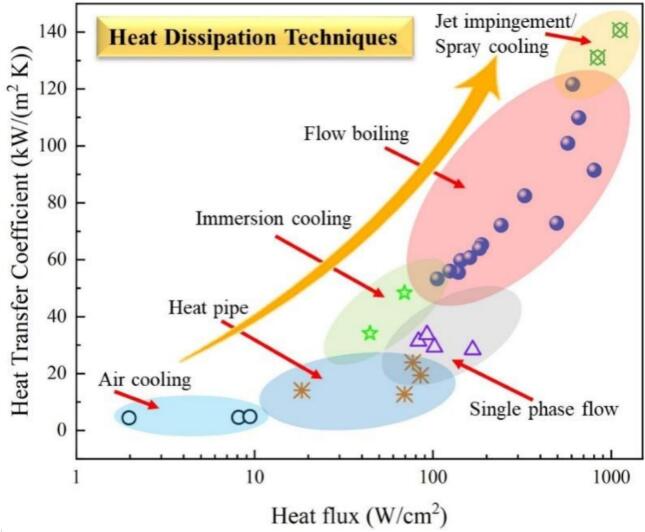
Heat transfer capability and performance distribution of different cooling techniques [3].

Traditional air and liquid cooling technologies can no longer meet the heat dissipation demands of modern high-heat-flux electronic devices [2]. In contrast, boiling heat transfer enhancement techniques demonstrate remarkable advantages [3], as illustrated in Fig. 1. Current heat transfer enhancement methods are mainly categorized into passive and active approaches: passive methods include microchannel structure optimization [4], surface treatment [5], and nanofluid applications [6,7], while active methods involve regulation techniques such as those using electromagnetic fields [8,9] and ultrasonic fields [10]. Among these, ultrasound-enhanced heat transfer technology has attracted significant attention due to its superior controllability [11]. By adjusting parameters such as the position, orientation, power, and frequency of ultrasonic transducers, this technology enables precise thermal regulation in specific regions, providing an effective solution to the thermal management challenges of microelectronic devices.

This review systematically summarizes the latest advances in ultrasound-enhanced heat transfer technology over the past five years, focusing on three core aspects: enhancement mechanisms, experimental studies, and numerical simulations. Section 2 elaborates on the fundamental mechanisms, including the thermal effect, acoustic cavitation, acoustic streaming, as well as acoustic fountain and atomization. Section 3 provides a comprehensive review of investigations in areas such as single-phase flow, pool boiling, forced convective boiling, heat exchanger performance, and bubble dynamics. Section 4 analyzes the effects of key parameters on heat transfer performance and their underlying mechanisms. Section 5 outlines future research challenges and development directions from the perspectives of multiscale coupling, energy efficiency, and adaptability under extreme conditions.

## 2 Mechanisms of ultrasound-enhanced heat transfer

**Fig. 2:**
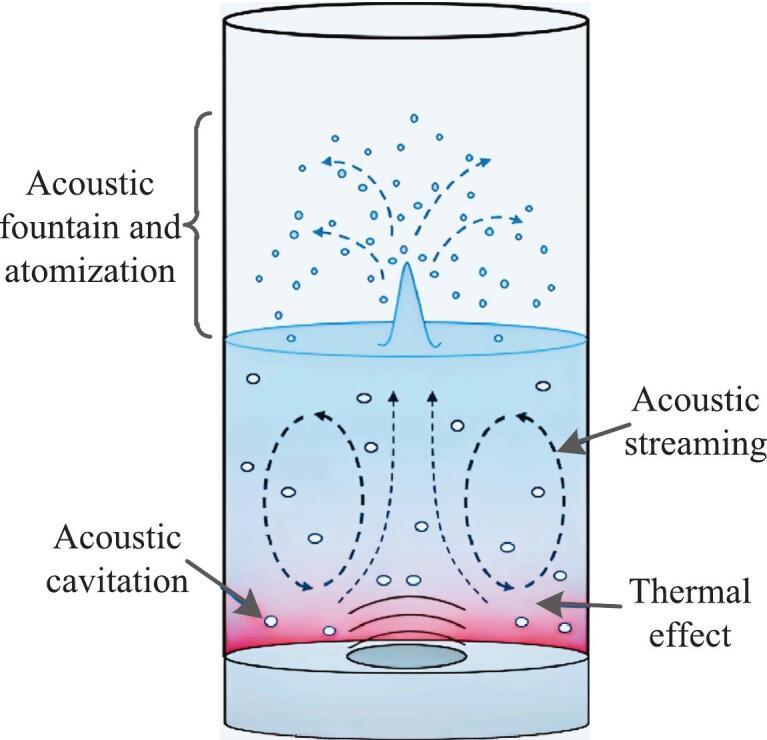
Phenomena and fluid motion resulting from the propagation of ultrasonic wave in liquid medium [37].

Ultrasonic waves can be classified into three categories based on frequency range: low-frequency (20–100 kHz), high-frequency (100 kHz-1 MHz), and ultra-high-frequency (1–10 MHz). Among these, low-frequency ultrasonic waves are also referred to as power ultrasonic waves due to their power characteristic. When these ultrasonic waves propagate through fluid, they generate various physical effects (Fig. 2), including the thermal effect, acoustic cavitation, acoustic streaming, and acoustic fountain and atomization.

### 2.1 Thermal effect

When ultrasonic waves propagated through a liquid, part of the acoustic energy was converted into heat, causing the liquid temperature to rise. This thermal effect occurred across all frequency ranges [12]. Notably, this temperature increase reduced vapor solubility and adversely affected vapor–liquid heat transfer processes. Therefore, in vapor–liquid heat transfer applications, cooling devices (such as fans, cooling jackets, cooling coils, or constant-temperature water baths) were often required to maintain isothermal conditions [13].

### 2.2 Acoustic cavitation

When ultrasonic waves propagated through liquid, they generated periodic compression-expansion effect. When the local pressure during the expansion phase fell below the saturated vapor pressure of the liquid, cavitation bubbles formed within the liquid. These bubbles first underwent an oscillatory growth phase and then collapsed violently once their size exceeded a critical threshold. The complete process was known as acoustic cavitation [14]. Based on bubble dynamic behavior, acoustic cavitation could be classified into two types: stable and transient [15]. Stable cavitation was characterized by bubbles oscillating persistently near an equilibrium size without collapsing, primarily generating microstreaming effect. This typically occurred under high-frequency ultrasound with smaller bubble sizes. In contrast, transient cavitation occurred under higher acoustic power, where bubbles underwent oscillatory growth followed by violent collapse, accompanied by intense shock waves and microjets.

The cavitation intensity was primarily governed by ultrasonic parameters. Acoustic power exhibited a positive correlation with cavitation intensity, while the cavitation threshold strongly depended on ultrasonic frequency. Studies have shown that low-frequency ultrasound more readily induced cavitation and generated larger bubbles (inversely proportional to frequency) [16]. Bulliard-Sauret et al. observed that 2 MHz ultrasound produced weaker transient cavitation [17], while Setareh et al. confirmed that transient cavitation played a dominant role in heat transfer enhancement [18]. The cavitation process triggered hydrodynamic effects such as microscale flows, shock waves, and microjets [19], all of which significantly enhanced heat transfer performance. Plesset and Chapman first established a theoretical model for bubble dynamics [20], and subsequent studies further elucidated the mechanism of ultrasound-enhanced heat transfer [[21], [22], [23], [24]]. Notably, bubble streams generated by acoustic cavitation disrupted boundary layer stability, thereby improving heat transfer efficiency [[25], [26], [27]]. Additionally, the cavitation effect under low-frequency ultrasound reduced the heating surface temperature by disrupting the stability of both the thermal and velocity boundary layers [28].

### 2.3 Acoustic streaming

When ultrasonic waves propagated through liquid, part of the acoustic energy was absorbed by the liquid, creating a momentum gradient that induced macroscopic convective flow, a phenomenon known as acoustic streaming [13]. Acoustic streaming was categorized into bulk streaming (caused by acoustic wave attenuation) and microstreaming (generated by cavitation bubble motion) [13], with typical velocity ranges of 1–100 cm/s [29]. By measuring the mass transfer rate in the diffusion boundary layer to indirectly reflect hydrodynamic characteristics, this finding indirectly supported the potential mechanism of ultrasound-enhanced heat transfer through disruption of the boundary layer [30]. While high-frequency ultrasound generated Eckart streaming, which created turbulent kinetic energy [31], its effect diminished with increasing flow velocity [17]. In contrast, ultrasound with low frequency and high intensity generated turbulence through cavitation effect, leading to superior heat transfer enhancement [32].

### 2.4 Acoustic fountain and atomization

Medium-to-high frequency ultrasonic waves induced unique acoustic fountain and atomization phenomena at vapor–liquid interfaces [33]. The mass flow rate in ultrasonic atomization increased with higher ultrasonic intensity and lower frequency. The fountain was formed at the liquid surface where the acoustic pressure exceeded atmospheric pressure, with its height primarily determined by the acoustic pressure at the transducer's center. While the dependence of atomized droplet number on ultrasonic frequency was small at constant ultrasonic intensity, higher frequencies yielded more atomized droplets for a given fountain surface area [34]. Localized acoustic pressures surpassing both cavitation and atomization thresholds suggested the coexistence of these phenomena in the acoustic fountain [35]. Further increasing ultrasonic intensity triggered atomization effect, a process jointly driven by capillary wave instability (producing uniform droplets) and microbubble collapse (generating dispersed droplets) [36].

Well-known ultrasonically induced effects such as acoustic cavitation and acoustic streaming were the two primary mechanisms for ultrasound-enhanced heat transfer in liquid [28,38,39]. It was very difficult to distinguish the influence of these effects since they often occurred simultaneously [40]. Among these, while low-frequency ultrasound (20–100 kHz) was dominated by acoustic cavitation, high-frequency ultrasound (>100 kHz) primarily exhibited acoustic streaming. Although thermal effect occurred during ultrasonic propagation, its energy conversion efficiency was relatively low [41]. Therefore, thermal effect was generally negligible and mainly applicable for calorimetric measurements of ultrasonic energy release in heat transfer systems.

## 3 Influence of ultrasound on heat transfer

### 3.1 Single-phase flow

**Fig. 3:**
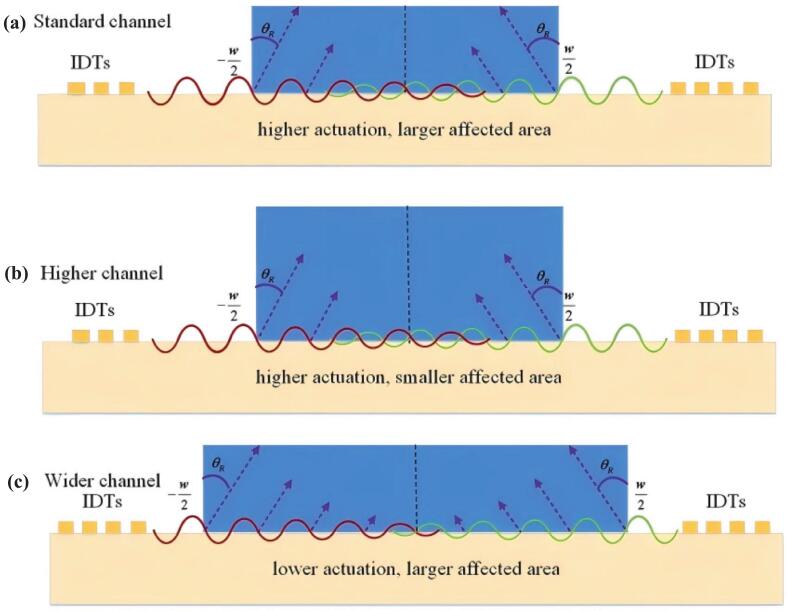
Plot of SSAW displacement along the bottom wall at three different microchannels [43].

The study proposed a heat transfer enhancement mechanism driven by acoustic body force-induced fluid motion in inhomogeneous fluids under ultrasonic standing waves, demonstrating that heat transfer efficiency was increased by up to 2.5 times compared to natural convection and 11.2 times compared to pure conduction when the standing wave was perpendicular to the heat transfer direction, while suppressing natural convection when the wave was parallel to it [42]. Perturbation theory studies revealed that standing surface acoustic wave (SSAW) in a straight microchannel heat sink created acoustic vortices through acoustic streaming and disrupted the thermal boundary layer, significantly improving heat transfer performance, as shown in Fig. 3, particularly demonstrating better thermal performance at shorter SSAW wavelengths and in narrower microchannels [43]. Furthermore, Das et al. established a theoretical model based on multiple-scale perturbation theory for surface acoustic wave (SAW)-driven acoustothermal heating of Newtonian fluids in microchannels, demonstrating that temperature rise depended linearly on acoustic energy density, with increased system size or SAW frequency enhancing energy conversion [44].

An elliptical acoustic focusing tank, utilizing acoustic interference and resonance properties to construct a focused sound field, enhanced local sound intensity and reduced energy dissipation, with experimental results showing a maximum heat transfer enhancement rate in natural convection [45]. Wan et al. also designed a cavity with an elliptical cross-section to create a focused ultrasonic field based on interference and standing wave criteria, which enhanced local sound intensity to improve heat transfer performance in both single-phase natural convection and subcooled boiling [46]. The heat transfer enhancement effect of 40 kHz ultrasound on FC-72 reached its optimum under quite low subcooled conditions [47]. For the spiral heater in a distilled water system, the application of ultrasonic waves led to significantly more uniform distributions of both velocity and temperature, with a maximum enhancement of 74 % in the natural convection heat transfer coefficient [48].

### 3.2 Pool boiling

**Fig. 4:**
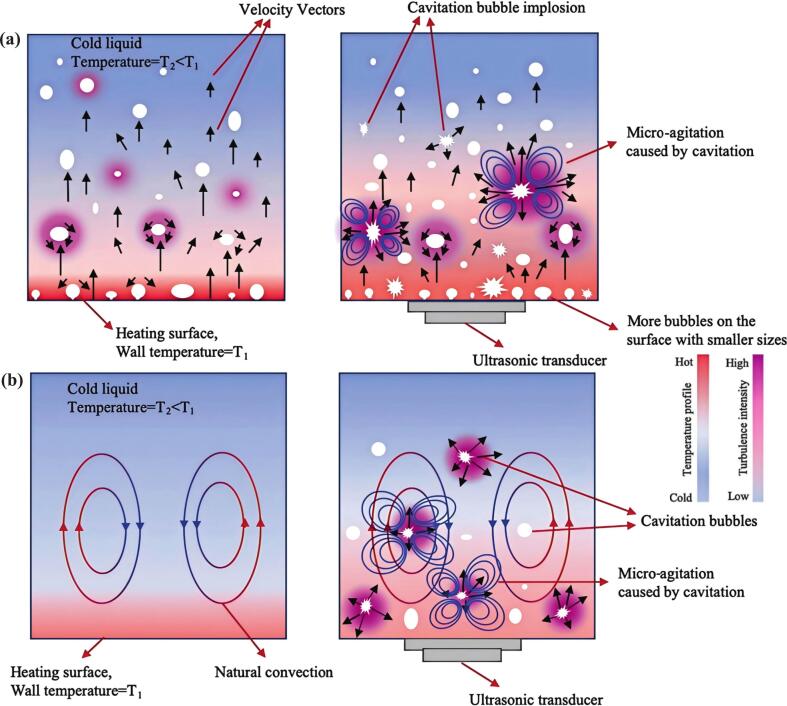
Schematic of (a) subcooled boiling and (b) convective heat transfer mechanisms with and without ultrasonic field propagation [55].

The ultrasound-enhanced effect on pool boiling primarily stemmed from its regulation mechanisms on bubble dynamics and boundary layers, as shown in Fig. 4a. Through cavitation effect, ultrasound could effectively activate nucleation sites and significantly increase bubble generation density. Simultaneously, acoustic pressure fluctuations facilitated bubble detachment, reduced detachment diameter, and effectively suppressed bubble coalescence. Furthermore, acoustic streaming enhanced liquid replenishment near the heating surface, delaying the occurrence of dry-out phenomena and thereby significantly improving the critical heat flux (CHF) [49]. It should be noted that excessive ultrasonic power might lead to excessive bubble fragmentation and reduce heat transfer efficiency. Optimizing bubble dynamics (e.g., reducing initial bubble size) enhanced heat transfer through cavitation-induced extreme conditions and acoustic streaming synergy, but required balancing radical yield and spatial distribution [50]. By coupling the Rayleigh-Plesset and energy equations, Hong and Song quantified the effects of nanobubble size and ultrasonic frequency on cavitation thresholds, confirming phase change as the dominant heat transfer mechanism [51]. Steady-state oscillation analysis demonstrated that the peak bubble temperature varied with the equilibrium radius, while cavitation activity was governed by the interplay between static pressure and ultrasonic frequency [52]. High-speed bubble imaging and spherical bubble statistical modeling successfully characterized the size distribution and hydroacoustic properties of bubble populations. By adjusting ultrasonic parameters and flow conditions, the cavitation effect was optimized to enhance heat transfer coefficient (HTC), specifically through the disruption of thermal boundary layers by microjets and shock waves [53]. The HTC distribution in focused ultrasonic cavities correlated with acoustic intensity, exhibiting higher acoustic streaming velocity and pronounced cavitation effects. Despite bubble clouds hindered heat transfer, cavitation collapse-induced high temperature, high pressure and microjets significantly enhanced thermal performance [54].

Acoustic field and acoustic streaming reduced bubble size and increased departure frequency, enhancing HTC and improving temperature uniformity in partial nucleate boiling regions, but had no significant effect in fully developed boiling due to large vapor columns [56]. While acoustic streaming markedly altered bubble behavior and near-wall flow, its effects diminished at high heat fluxes or large gaps due to competition between bubble-induced convection and acoustic streaming [57]. Studies showed that ultrasound enhanced HTC and reduced surface temperature via acoustic streaming, particularly at low heat fluxes, while particle image velocimetry (PIV) and high-speed imaging confirmed its regulation of bubble behavior and fluid mixing [58], providing novel solutions for electronic cooling [[59], [60], [61]]. The phase field method (PFM) coupled with thermo-viscous acoustic simulations revealed that SAW enhanced downward-facing boiling through vortex-induced bubble detachment and removal, disrupting thermal boundary layers and intensifying phase-change heat transfer [62].

Despite its potential, ultrasound-enhanced pool boiling faced critical challenges: including energy attenuation which required high-power or resonance conditions, bubble-induced acoustic attenuation which led to failure at high heat fluxes, and multi-factor synergy (e.g., subcooling, pressure, and surface morphology) which constrained the enhancement efficacy [63].

### 3.3 Forced convective boiling

The ultrasonic cavitation effect significantly enhanced the HTC by increasing nucleation density, advancing the onset of nucleate boiling (ONB), and intensifying bubble collisions, but the enhancement ratios diminished at high heat fluxes due to flow pattern transitions [45,[64], [65], [66], [67]]. Low-frequency ultrasound (25 kHz) enhanced turbulent kinetic energy via cavitation (bubble oscillation and collapse), disrupting boundary layers and improving HTC especially at high flow rates. Ultra-high-frequency ultrasound (2 MHz) generated global mixing through acoustic streaming, showing stronger enhancement at low flow rates but reduced effects with increasing flow velocity. Frequency selection needed to be optimized based on flow conditions for optimal heat transfer enhancement [32], with low-frequency results consistent with [68]. Ultrasonic cavitation activated abundant vapor embryos to reduce wall superheat for ONB, while high-frequency pressure fluctuations enhanced heat transfer by promoting bubble nucleation, growth, and detachment [69]. Xu et al. demonstrated that parametric resonance-induced non-inertial cavitation generated stable periodic flow oscillations to optimize heat transfer [70], and correlated liquid film thickness with surface wave vibration to elucidate acoustic streaming-enhanced mass transfer near vapor–liquid interfaces [71].

Ultrasound enhanced forced convective boiling by regulating bubble dynamics, strengthening acoustic streaming-induced secondary flow, and stabilizing vapor–liquid flow (Fig. 4b), with mechanisms varying by boiling conditions [63]. Ultra-high-frequency ultrasound (2 MHz) improved near-wall heat transfer via non-cavitation amplified fluid vibrations [17]. Setareh et al. demonstrated that ultrasonic field (mainly via acoustic streaming) generated secondary circulations that enhanced fluid mixing and heat transfer by up to 87 % at low Reynolds numbers (*Re*), though the enhancement diminished with increasing Reynolds number [72]. Poncet et al. revealed that both low-frequency cavitation and high-frequency acoustic streaming enhanced convective heat transfer in laminar/turbulent flows by disrupting thermal boundary layers, with effects diminishing at higher *Re* and correlating positively with initial boundary layer thickness [73]. Non-dissipative acoustic body forces from light-induced temperature gradients drove liquid acoustic streaming at velocities nearly 100 times higher than boundary-driven Rayleigh streaming and Rayleigh-Benard convection, enabling modular microflow control and enhanced heat transfer [74]. Time-scale separation simulations confirmed that low-frequency high-intensity sound waves generated strong acoustic streaming disturbances inside and outside the thermal viscous boundary layer of heat exchanger tube, significantly enhancing heat transfer [75]. SAW suppressed flow blockage and instability by fragmenting bubbles and enhancing fluid mixing. In addition, the synergy between acoustic radiation force and buoyancy promoted bubble detachment and secondary bubble formation [76].

The combined action of low-frequency cavitation and high-frequency acoustic streaming generated superior heat transfer enhancement compared to single-frequency ultrasound, which was linked to greater induced fluid turbulence [77]. Equivalent ultrasound propagation methods with turbulence models confirmed the enhancement effect of single/dual transducers at specific positions, which originated from acoustic streaming-disrupted boundary layers and cavitation-induced turbulence and microjets [78]. Altay et al. identified the minimum bubble size required for acoustic field effects on clusters [79]. Studies integrating user-defined functions (UDF) based on dynamic mesh control with cavitation model revealed that acoustic streaming vortices and microjets drove temperature field homogenization [80].

### 3.4 Heat exchanger

Heat exchangers, as widely utilized heat transfer equipment in industrial applications, played a significant role in heating, refrigeration, chemical processes, and thermal energy recovery [81,82]. Research has demonstrated that ultrasonic technology could effectively enhance heat exchanger performance, with the enhancement mechanisms primarily involving acoustic cavitation, acoustic streaming disturbance, and thermal boundary layer disruption. Therefore, active heat transfer enhancement mechanisms have garnered widespread attention for heat exchanger optimization [83,84].

**Fig. 5:**
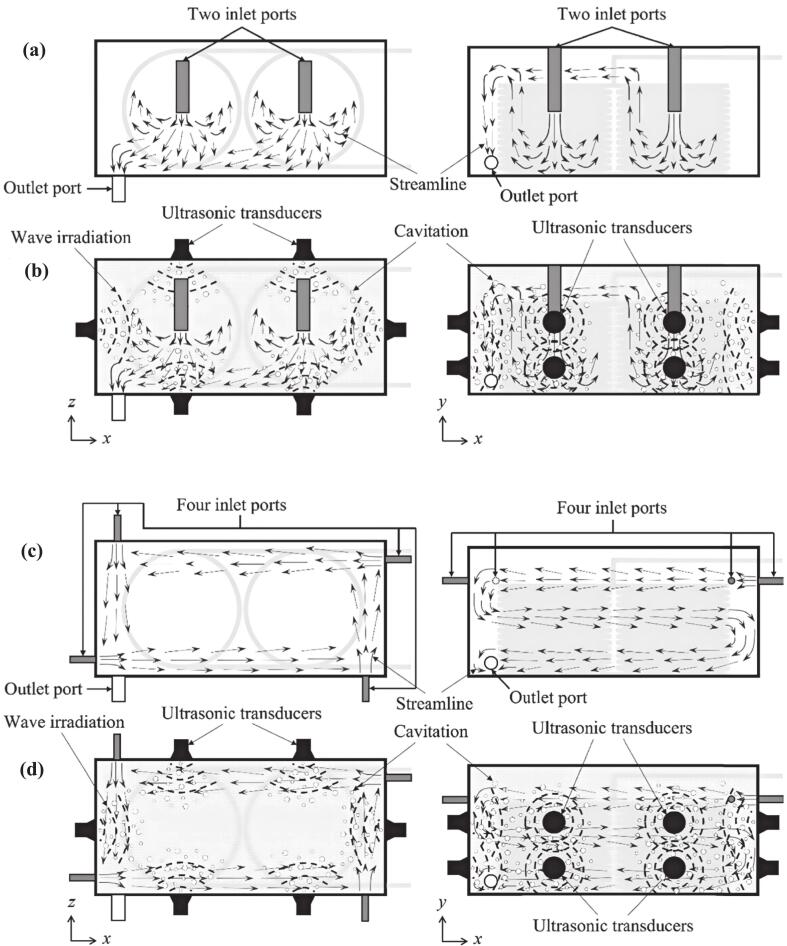
Flow pattern and mechanism of heat exchanger in configuration 1 (a) with and (b) without ultrasound and in configuration 2 (c) with and (d) without ultrasound [38].

In a double vertical coil and shell heat exchanger, as shown in Fig. 5, applying 28 kHz ultrasound along with an optimized flow configuration was found to enhance the shell-side Nusselt number and reduce pressure drop. The improvement in heat transfer performance exceeded the amount of wave energy supplied [38]. Azimy et al. directly mounted ultrasonic transducers in contact with the fluid, unlike in prior methods. Ultrasound significantly increased the HTC by intensifying turbulence and cavitation. The enhancement grew with higher ultrasonic power but diminished with increased flow rate or heater power [85]. It also suppressed nanoparticle sedimentation and agglomeration by strengthening Brownian motion and elevating Zeta potential (stability) [86]. Extensive studies demonstrated a positive correlation between ultrasonic power and enhancement factor [[87], [88], [89]], while fluid flow velocity exhibited an inverse relationship [18,68,72], with the enhancement effect being further influenced by fluid properties and flow patterns [90]. In counter flow plate heat exchanger, ultrasound could effectively enhance enthalpy recovery efficiency [91]. Ultrasound significantly enhanced heat and mass transfer efficiency in droplet cooling and freezing processes by generating microbubbles through cavitation and intensifying surface renewal [92].

In fin-and-flat tube heat exchangers, the enhancement was more pronounced under low flow rate, ambient temperature, and air passing velocity [93]. Ultrasound at 40 kHz significantly improved the HTC in double-pipe heat exchangers (DPHX) by disrupting the thermal boundary layer and enhancing fluid mixing through acoustic streaming, cavitation, and micro-convection. A maximum heat transfer improvement of 104 % was achieved at an inlet temperature of 60 ℃ and an ultrasonic power of 240 W [94]. Prediction models using the Group Method of Data Handling (GMDH) and Response Surface Methodology (RSM) accurately evaluated fin-and-tube heat exchanger (FTHX) performance based on input parameters such as inlet temperature, flow rate, air velocity, and ultrasonic power level, with the GMDH model slightly outperforming the RSM model [95]. Esfandyari et al. employed an artificial neural network (ANN) and adaptive neuro-fuzzy inference system (ANFIS) integrated with particle swarm optimization (PSO) to predict the heat transfer rate, Nusselt number, number of transfer units (NTU), and effectiveness of a double-pipe counter-flow heat exchanger [84].

The aforementioned studies demonstrated that ultrasound-enhanced heat exchanger performance was primarily governed by the combined effects of heat exchanger configurations, thermal–hydraulic parameters, ultrasonic parameters, and working fluid properties [38,68,85,90,94].

### 3.5 Bubble characteristics

**Fig. 6:**
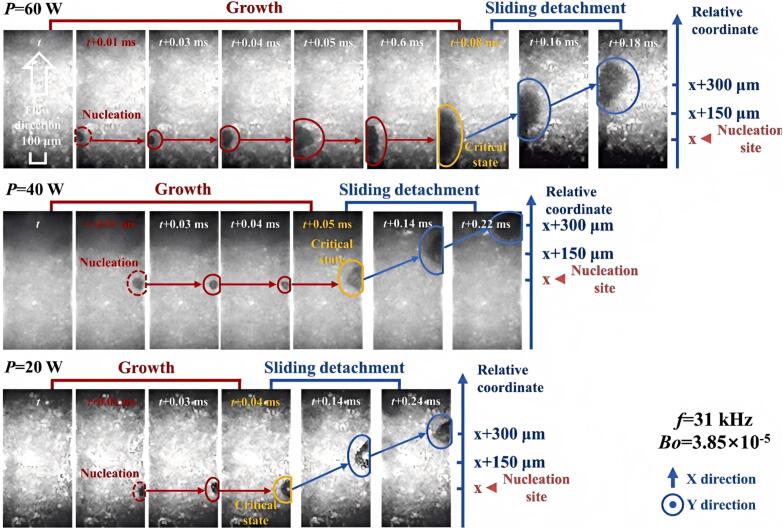
Bubble behavior under different ultrasonic powers [102].

**Fig. 7:**
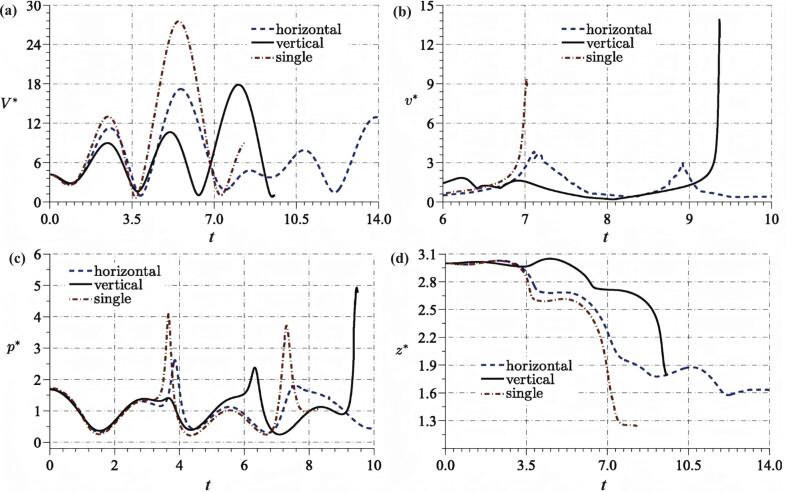
Bubble parameters and induced field pressure for different bubble configurations in a standing ultrasonic field. Time histories of (a) the volume of the bubble near boundary, (b) the jet velocity, (c) the pressure at the point near the boundary (0, 0, 0.1) for the three cases and (d) translation of the bubble center in the z^∗^ direction [110].

Ultrasound regulated bubble dynamics, which served as a key mechanism for enhancing heat transfer performance and improving critical heat flux (CHF), demonstrating significant application value in fields such as microelectronic device cooling [67,76,96,97]. Heat transfer was enhanced through SAW-driven acoustic streaming and bubble dynamics control (vibration/splitting/detachment), with the core mechanism identified as the synergistic disruption of the thermal boundary layer and active bubble behavior manipulation [76]. The acoustic field induced capillary waves on vapor bubble surfaces, causing bubble contact line contraction and detachment from the heating surface, while acoustic radiation pressure further promoted bubble detachment and suppressed the transition to film boiling, thereby enhancing CHF [98]. Acoustic pressure amplification in a narrow slit deformed, oscillated and self-assembled microbubbles, thereby improving HTC [99]. The ultrasonic field enhanced heat transfer by inducing capillary waves on bubble surfaces and increasing both detachment frequency and motion velocity of bubbles, which destabilized vapor–liquid two-phase flow and promoted fluid mixing [100]. The ultrasonic field generated primary and secondary Bjerknes forces. The former drove bubbles to slide rapidly along the heating wall (sweeping effect), while the latter caused attraction or repulsion between bubbles (cutting effect), significantly shortening bubble growth time and increasing detachment velocity (Fig. 7) [101,102]. In Fig. 6, Boiling number *Bo* = *q*/(*Gh*_lg_), where *q* is the heat flux, W/m^2^; *G* is the mass flux, kg/(m^2^ s); *h*_lg_ is the latent heat of vaporization, J/kg.

Heat transfer was enhanced through acoustic cavitation (bubble oscillation, clustering, and collapse) and self-organized structures induced by secondary Bjerknes forces, with the core mechanism involving synergistic effects of localized energy concentration and microflows [103,104]. Through high-speed imaging and a mirror principle model, Liu et al. observed that bubble collapse generated localized high temperature and pressure, inducing micro-convection. Bubble aggregation and motion altered local flow conditions and the thermal boundary layer, while bubble vibration and secondary acoustic radiation disturbed the surrounding liquid, collectively enhancing heat transfer [105]. The interaction between bubbles via radiated pressure waves affected their radial pulsations, altering expansion ratios and vibrational behavior, which modulated the local flow field and energy distribution [106]. Bubble radial pulsation, translation, and deformation influenced heat transfer by altering local flow field structures and energy distribution, while Bjerknes forces between bubbles further regulated their motion and deformation [107]. Similar conclusions were reported by Ma et al., who reported that nonspherical bubble distortion influenced inter-bubble interaction by altering the strength and direction of the secondary Bjerknes force [108]. Studies also showed that the equilibrium bubble radius and the distance between wall and bubbles significantly affected the compression ratio [109], the orientation of bubble pairs modulated jet intensity toward the boundary (Fig. 7) [110], and the initial bubble radius and the normalized standoff distance strongly determined the collapse time and liquid jet speed of acoustic cavitation bubbles [111]. Han et al. revealed the regulatory mechanisms of mass and heat transfer on near-wall dual-bubble cavitation behavior through a novel model coupling the modified Keller-Miksis equation with the Noble-Abel Stiffened Gas (NASG) equation of state [112]. Dehane et al. found that thermal conduction dominated the bubble internal energy balance at lower acoustic amplitudes, while mass transport became more significant at higher acoustic amplitudes, and the optimum bubble radius for the maximal response shifted toward smaller values with increasing frequency or amplitude [[113], [114], [115]]. Peng et al. found that increased liquid temperature altered the vapor amount inside bubbles, changing the relative magnitude of the compression force and cushion effect, which caused the collapse intensity to first rise and then fall, peaking at an optimum temperature [116]. Altay et al. reported that surface roughness reduced bubble size and weakened cavitation intensity by affecting wave propagation and enhancing scattering, leading to a lower and more rapidly stabilized pool temperature [79].

At high flow rates, acoustic field limited influence on single-phase flow heat transfer. In boiling heat transfer, heat transfer enhancement could even be inhibited under certain fluid temperature due to the strong pressure pulse formed by rupture of ultrasonic cavitation bubbles [117]. In the field of ultrasonic technology, the energy conversion process still lacked systematic theoretical analysis and experimental validation [81]. In addition, most research on energy efficiency evaluation of heat exchangers mainly focused on passive enhancement techniques [118], and related explorations in microchannel heat transfer systems were relatively insufficient [119].

## 4 Parametric studies related to ultrasound-enhanced heat transfer

### 4.1 Ultrasonic frequency

Heat transfer enhancement was found to be greater at 21 kHz than at 45 kHz, as most of the tube's lower surface was located in the antinode area, promoting bubble dynamics [120]. Although 33 kHz waves provided better heat transfer enhancement compared to 40 kHz, they also induced higher system pressure loss [39]. Other studies have shown that low-frequency ultrasound was reported to cause only slight increases in system pressure loss [41,121]. Ultrasound at 40 kHz and 5 cm distance achieved a maximum heat transfer enhancement of 82.4 %, while 120 kHz performed best at 10 cm and 15 cm (74.2 % and 59.7 %, respectively) [122]. Low-frequency (25 kHz) and ultra-high-frequency (2 MHz) ultrasound exhibited distinct enhancement mechanisms: the effect of acoustic streaming (2 MHz) decreased with increasing flow rate, consistent with reduced turbulent kinetic energy, whereas the cavitation effect (25 kHz) strengthened due to the larger relative size of acoustic bubbles with respect to the laminar boundary layer [32]. Combined low-and-high-frequency ultrasound enhanced heat transfer more effectively than single-frequency ultrasound by generating greater turbulence in the water flow. However, the induced turbulence was not uniformly distributed, and the synergistic effect primarily relied on turbulence generation along the heating wall [77]. It was shown that 2 MHz ultrasound strongly altered the flow pattern in the channel due to abrupt changes in flow direction and an increased turbulence rate [17]. Additionally, heat transfer improvement was found to decrease with increasing ultrasonic frequency at constant heat flux, as higher frequencies promoted bubble coalescence over heat absorption during growth [67].

**Fig. 8:**
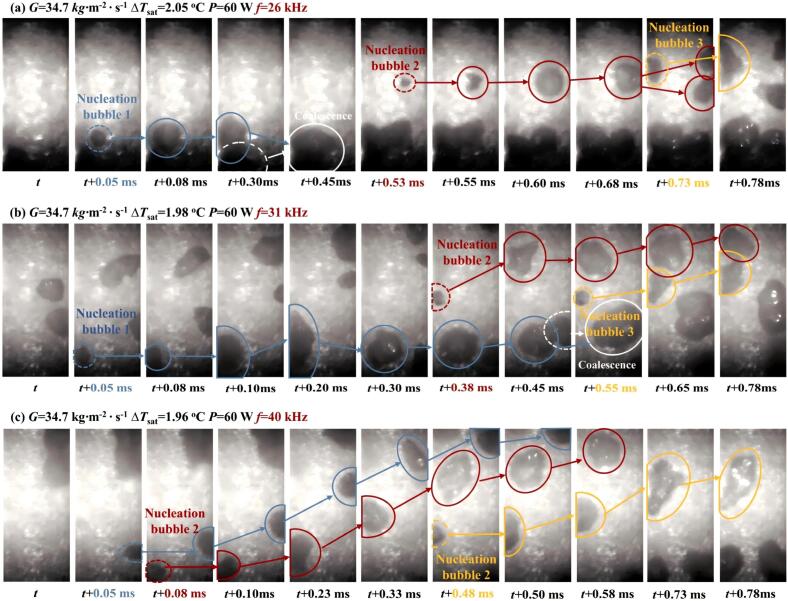
Bubble nucleation at the bottom wall with different ultrasonic frequencies [69].

The heat transfer mechanism depended on the relationship between ultrasonic frequency and bubble resonance frequency. Analysis of dual-bubble and high-pressure cases showed that the process could be approximated as adiabatic under high-pressure conditions with ultrasonic frequency far from the resonance frequency [123]. Guo et al. found that the quantities of nucleated bubbles were similar across frequencies, and vapor bubble generation showed low sensitivity to frequency changes, as shown in Fig. 8. Although frequency and amplitude were inversely related at a given power, their product remained approximately constant, resulting in stable ultrasonic pressure amplitude and no significant effect on wall superheat at ONB [69]. The ambient bubble radius range narrowed with increasing ultrasonic frequency, and heat exchange at the bubble interface was identified as the primary mechanism in the internal energy balance [113]. When ultrasonic intensity was large enough, lower frequencies improved the overall performance of the shell and tube heat exchangers with twisted tape (STHXTT) due to enhanced ultrasonic cavitation, thermal, and vibration effects under longer acoustic wave period [124]. By comparing the results from a variety of studies, Phelan et al. concluded that lower ultrasonic frequencies enhanced forced convection and subcooled boiling more effectively than higher frequencies, particularly within the tens of kHz range, whereas higher frequencies yielded greater improvement in natural convection [125].

### 4.2 Ultrasonic power

Li et al. demonstrated that heat transfer performance in both smooth and screwed tubes was more effective at 90 W than at 30 W across all heat flux ranges [120]. Wan et al. found that the ultrasound enhancement ratios for different tube types decreased with increasing ultrasonic power [126]. It was shown that heat transfer enhancement increased with ultrasonic power at constant heating power and flow rate, as the maximum cavitation bubble radius (*R*_max_) was proportional to ultrasonic power and inversely proportional to ultrasonic frequency. Larger *R*_max_ resulted in greater disruption during bubble collapse, leading to heat transfer improvement [127]. High ultrasonic power significantly increased the number of nucleated embryos on the heating surface at low wall superheat at ONB, which was attributed to the dominance of ultrasonic power in the ultrasonic pressure that dominated cavitation [69]. Increasing ultrasonic power directly increased the HTC [85,86,128,129] and enhanced the convection coefficient more noticeably at lower flow rates [94].

### 4.3 Transducer configuration

**Fig. 9:**
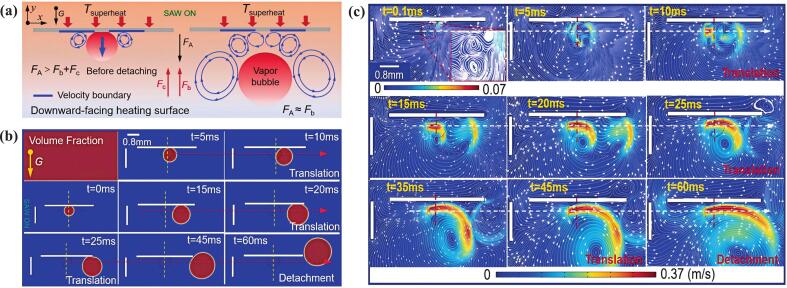
Evolution process of downward heating surface under transverse SAW excitation (a) mechanism diagram of the effect of longitudinal SAW excitation on the bubble behavior, (b) snapshots of bubble growth and (c) velocity field and streamline of second-order field [62].

All ultrasonic combinations significantly increased heat transfer in both the entrance and fully developed regions compared to no ultrasound. However, less enhancement occurred when transducers were placed near the channel entrance due to interference from unstable entrance flow inside the tube. Additionally, heat transfer improvement did not absolutely rise with the number of transducers, for reasons that remained unclear [130]. Different transducer placement types all enhanced the heat transfer, especially at lower volumetric airflow rates and higher ultrasonic power levels, though the enhancement rate varied by placement type [129]. Ultrasound significantly augmented flow boiling heat transfer, although the intensification effect weakened as heat flux increased. Moreover, the enhancement efficiency rose with larger radiation angles due to the resulting increase in ultrasonic pressure amplitude [67]. Heat transfer enhancement was observed to decrease with an increase in the distance from and the incident angle on the heater. The maximum enhancement ratio occurred when the heating surface was aligned with the transducer center [122]. Acoustic cavitation with ultrasound reduced the heating surface temperature, and the local Nusselt number increased when the transducer was placed in the middle of the duct. Ultrasonic effects were transported along with the flow when the transducer was upstream, and waves released downstream still affected heat transfer on the cylindrical surface at low *Re* [131]. Under longitudinal surface acoustic wave (SAW) excitation, vortices formed at the bubble root due to acoustic streaming, promoting bubble detachment while inhibiting lateral growth and movement of the bubble. Thus, the bubble could be suspended in the liquid, almost statically. Transverse SAW generated vortices that produced translational thrust on the bubble, causing lateral motion and eventual departure from the surface. Under superimposed SAWs excitation, the transverse SAW disrupted the vertical flow balance around the bubble initially established by the longitudinal SAW, and the unbalanced vortices destroyed the thermal boundary layer and weakened viscous effects. Fig. 9 shows the evolution of a downward heating surface under transverse SAW excitation [62]. When ultrasound was applied at the outlet, its propagation direction opposed the fluid flow, resulting in weaker cavitation and poorer heat transfer performance compared to application at the inlet. Horizontal transducer placement led to small vertical amplitude, reduced cavitation, and less effective heat transfer enhancement [124].

### 4.4 Flow rate

Ultrasound was found to effectively enhance heat transfer in turbulent pipe flow within the *Re* range of 10000–15000. Beyond this range, the enhancement capability diminished, and the *Nu*_w_/*Nu*_o_ ratio (Nusselt number with ultrasonic waves/Nusselt number without ultrasonic waves) fell below 1, as higher flow inertial force convected high-temperature water downstream, obstructing heat convection from the heating surface and reducing heat transfer [39]. At low *Re* and constant ultra-high-frequency ultrasound (2.8 MHz), acoustic streaming was found to disrupt the wall boundary layer and enhance fluid-wall heat transfer. As *Re* increased, flow velocity gradually dominated the heat transfer process, and ultrasound primarily improved the synergy between the flow and temperature fields [10]. Multiple studies indicated that the effect of ultrasound on heat transfer enhancement diminished with increasing flow rate and other driving forces [127,128]. Experimental results showed that increasing *Re* reduced the effect of acoustic streaming relative to fluid flow on heat transfer and the flow field. The cross-flow generated by acoustic streaming circulations was responsible for heat transfer enhancement [72]. The HTC ratio decreased with rising flow rate, as the turbulence induced by ultrasound diminished compared to flow inertia [85]. Experimental results showed that 25 kHz ultrasound enhanced heat transfer from laminar to turbulent patterns by disturbing the thermal boundary layer through acoustic cavitation. The enhancement factor decreased with increasing *Re* until reaching an asymptote in the turbulent pattern. For 2 MHz ultrasound, acoustic streaming improved convective effects within the flow, enhancing heat transfer; however, the enhancement factor declined as *Re* rose and ceased entirely beyond *Re* = 7500 [73]. Reducing the flow rate, ambient temperature, and air passing velocity on the heat exchanger increased the effects of ultrasonic excitation [93].

Inworn and Chaiworapuek revealed that ultrasonic waves propagating downward enhanced heat transfer in a region that moved downstream and vanished at the highest free-stream velocity. The ultrasonic beam was convected by the mainstream, and its inclination changed with free-stream velocity. The transducer produced an acoustic beam resembling the jet flow, which transported cooler water from the upper flow to replace heated near-wall water, reducing surface temperature and increasing the Nusselt number [132]. The thermal response of the microchannel accelerated with ultrasound. Cavitation and acoustic streaming effects disturbed the near-wall flow boundary layer, strengthening heat transfer. Lower *Re* resulted in better heat transfer enhancement, which was attributed to the fact that the small *Re* corresponded to a large energy per unit time exerted by adding ultrasound. Tests showed that the pressure drop primarily came from viscous loss in the channel, and ultrasound had no negative impact on it [133]. Ultrasound significantly enhanced heat transfer, reduced flow resistance, and improved overall performance. However, as the *Re* increased, the reduction in resistance and the overall performance improvement weakened [124].

### 4.5 Heat flux

Heat transfer enhancement ratios were generally lower under higher heat flux conditions compared to lower heat flux or lower Rayleigh number conditions. This finding aligned with previous literature [134] reporting that enhancement ratios in natural convection were higher than those in subcooled boiling [64]. For nucleate boiling on a small plain heater, ultrasound with amplitudes of 5–10 µm enhanced heat transfer at low heat flux. Visualization showed that acoustic streaming contributed more at higher amplitudes, while acoustic forces dominated at lower amplitudes. As heat flux increased, the ultrasonic effect diminished due to the violent growth and departure of vapor bubbles on the heating surface [135]. Ultrasonic enhancement exhibited limited efficacy in saturated boiling, primarily attributable to strong acoustic attenuation by bubbles at high heat fluxes [136,137]. At low heat fluxes (single-phase natural convection), the heat transfer enhancement factor slightly decreased with greater distance between the transducer and heating rod. However, the enhancement factor increased with distance at high heat fluxes (subcooled nucleate boiling). In subcooled nucleate boiling, the mechanism of heat transfer depended not only on acoustic streaming but also on the boiling pattern: more bubbles formed on the rod surface, moved erratically, mixed the surrounding water, and brought cooler water to the rod, thereby enhancing the rate of heat transfer [138].

For the screwed and finned tube, the HTC exhibited a “wave-like” trend versus heat flux: it rose at ONB, fell with further heat flux increase, and rose again at very high heat flux. Ultrasound caused bubbles on structured tubes to coalesce and grow, which resulted in a decrease in HTC. However, high heat flux and acoustic streaming promoted vigorous bubble motion and departure, leading to a rise in HTC again [120]. Increasing the heat flux increased HTC, and decreased the effect of ultrasonic waves on HTC [85]. Ultra-high-frequency ultrasound enhanced heat transfer on the vertical heating surface by disturbing the near-wall flow, especially at lower heat fluxes. At higher heat fluxes, the interaction with the waves became more complex, increasing velocities but limiting streaming coverage in the lower region of the heating surface [58]. Ultrasound prominently enhanced heat transfer at low heat fluxes, but its effect weakened as heat flux increased due to the transition from bubbly to confined bubbly flow. After the flow pattern transitioned to elongating confined bubbly flow, heat transfer deteriorated when heat flux was further increased [67]. While ultrasound hindered the saturated boiling heat transfer of the smooth and two-dimensional finned tubes, the ultrasound enhancement ratio for the three-dimensional finned tube was negative at low heat flux but positive at high heat flux [126].

### 4.6 Subcooling

Under higher heat flux conditions, the subcooling effect induced by ultrasonic vibration became less significant due to intense bulk nucleate boiling [64]. In the transition boiling region, high-amplitude ultrasound promoted the occurrence of microbubble emission boiling (MEB) at a subcooling of 15 K, where it normally did not occur. However, its effects on bubble behavior and HTC became insignificant when stable MEB was established at 21–40 K subcooling. As subcooling increased to 50–60 K, the unstable vapor film was sustained for a very short time before being broken up and condensed under acoustic streaming and capillary waves, leading to further heat transfer enhancement [135]. Horiuchi et al. investigated non-uniform spatial behaviors in MEB and found that the linear correlation between heater power input and surface heat flux broke down during MEB transition [139]. The MEB phenomenon was characterized by an anomalous heat flux exceeding the CHF at a critical subcooling level [[140], [141], [142]]. This process involved coupled mechanisms of instantaneous bubble collapse and micron-scale bubble cluster ejection [143], exhibiting physical similarities to microbubble dynamics in quenching boiling. At higher temperatures, the fluid temperature rose, providing greater thermal conductivity and lower viscosity, which resulted in an increased HTC [86]. Jalali et al. revealed that variations in the hot fluid inlet temperature did not significantly influence the HTC improvement [94]. The enhancement effect of ultrasound on heat transfer and CHF depended considerably on subcooling and boiling space height. In the nucleate boiling pattern, acoustic streaming altered upward liquid flow and vortex structures related to bubble dynamics (growth, departure and rising). As heat flux increased, the bubble-induced convection accelerated and competed with the acoustic streaming near the heating surface, weakening the ultrasonic effect on heat transfer. In the transition boiling pattern, the effects of ultrasonic waves on heat transfer of MEB depended on the competition between acoustic streaming and vapor-film-induced oscillating flow, with enhancement occurring when acoustic streaming dominated near the wall [57]. Yu et al. found that inlet subcooling had no significant influence on the intensified effects of ultrasound [67].

### 4.7 Nanofluids

**Fig. 10:**
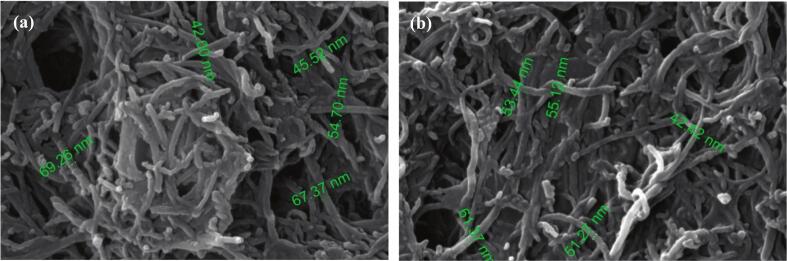
FE-SEM image of nanofluid after using in heat exchanger: (a) without ultrasonic waves, (b) with ultrasonic waves [86].

Nanofluid, as a potential working fluid, combined with ultrasound demonstrated significant advantage of heat transfer enhancement [86,144,145]. Although surfactants addition reduced surface tension, their deposition on the heater limited HTC improvement, causing the surfactant to be ineffective in increasing the HTC. Therefore, surfactant addition only increased HTC at low heat fluxes. Ultrasonic waves dramatically increased HTC across all heat fluxes without additives, by enlarging bubble departure diameter and reducing fluid viscosity via cavitation [145]. In pulsating heat pipes (PHP), nanofluid assisted with ultrasound intensified working fluid oscillation and stabilized the HTC trend of acetone-filled PHP under varying heating power [146]. The increasing ultrasonic power also increased the instantaneous velocity and driving force of the fluid, shortened startup time, and promoted nanoparticle dispersion via microjets from cavitation bubble collapse and oscillation, thereby improving the heat transfer performance of high concentration nanofluid PHP [147]. Ultrasonic vibration could reduce the tube pressure drop, especially at low flow rates and high nanoparticle volume fractions. Increasing nanoparticle volume fraction enhanced heat convection under ultrasonic waves, with acoustic cavitation identified as the primary mechanism. However, higher *Re* hindered acoustic bubble production, and at very high *Re*, eliminated the positive effects of ultrasound [90]. The use of ultrasonic transducers increased the HTC and improved nanofluid stability (Fig. 10). The enhancement effect of ultrasound grew with higher nanoparticle concentration but diminished with increased flow rate [86].

Ultrasonic waves could enhance heat transfer in the water-Al_2_O_3_ nanofluid, but the heat transfer efficiency decreased with increasing heat flux. In subcooled boiling, ultrasound improved heat transfer in the nanofluids at 50, 70, and 80 ℃, but inhibited it at 60 ℃ due to the strong pressure pulse from cavitation bubble collapse. Therefore, controlling the main body temperature during heat transfer was not suitable at temperatures where ultrasonic cavitation was most likely to occur. In saturated boiling, ultrasound initially strengthened HTC as heat flux increased, but the heat transfer enhancement gradually declined and became less effective at high heat fluxes [148]. The surface temperature rose with higher Al_2_O_3_ volume fraction due to changes in thermophysical properties but dropped rapidly after ultrasound application. Ultrasound showed a stronger cooling effect in Al_2_O_3_ at φ = 0.8 % than at φ = 6.1 %. Denser nanoparticles in higher concentration fluids attenuated wave propagation, reducing wave intensity and thermal enhancement capability. Lower fluid density and viscosity strengthened the heat transfer enhancement by acoustic cavitation. However, localized hotspots also occurred during ultrasonication due to bubble structures shielding the ultrasonic field on the heat transfer surface. On the one hand, cavitation bubbles thickened the thermal boundary layer, making heat exchange more difficult. On the other hand, in nanofluid systems, the transient and severe collapse of cavitation bubbles caused dramatic temperature rises, and the short heat exchange period was insufficient for cooling [149]. Hence, the temperature of hot vapor was extremely high, resulting in persistent localized hotspots [150].

The synergy originated from enhanced Brownian motion of small particles boosting heat transfer, coupled with the use of ultrasound to minimize particle deposition [151]. Hedeshi et al. demonstrated that adding nanoparticles and applying ultrasonic vibrations were particularly effective at higher inlet temperatures and nanofluid concentrations. Nanoparticles performed better at high flow rates, while ultrasound was more prominent at low flow rates. They concluded that impurities such as nanoparticles increased the probability of acoustic bubble formation [152]. The relationship between HTC and ultrasonic power, nanofluid concentration, and fluid temperature could be predicted using an Artificial Neural Network (ANN) with 15 hidden neurons and a ''trainbr'' training algorithm [153].

### 4.8 Channel structure

Ultrasound enhanced heat transfer more effectively in tubes with surface structures than in smooth tubes and reduced the performance differences between various structures [120]. Ultrasound made bubbles on sintered porous surfaces more susceptible to acoustic streaming and reduced their departure diameters. Alternating acoustic pressure activated additional vaporization cores, but the ultrasound-enhanced heat transfer effect was restricted with increasing heat flux [137]. Coupling metallic foam with ultrasound slightly increased the HTC in partially porous channel compared to a flow without ultrasound, but showed no enhancement in fully porous ones due to the impedance of wave propagation medium, which attenuated the acoustic streaming effect [154]. Ultrasonic enhancement provided limited benefits for saturated boiling and even hindered heat transfer in smooth and two-dimensional finned tubes. At 21 kHz, the three-dimensional finned tube showed a negative ultrasound enhancement ratio at low heat flux but a positive one at high heat flux. The three-dimensional structure improved heat transfer by reducing wall-bubble interaction and generating liquid vortex flow through ultrasonic propagation along the intersected grooves, which enhanced bubble stretching and contraction [126].

**Fig. 11:**
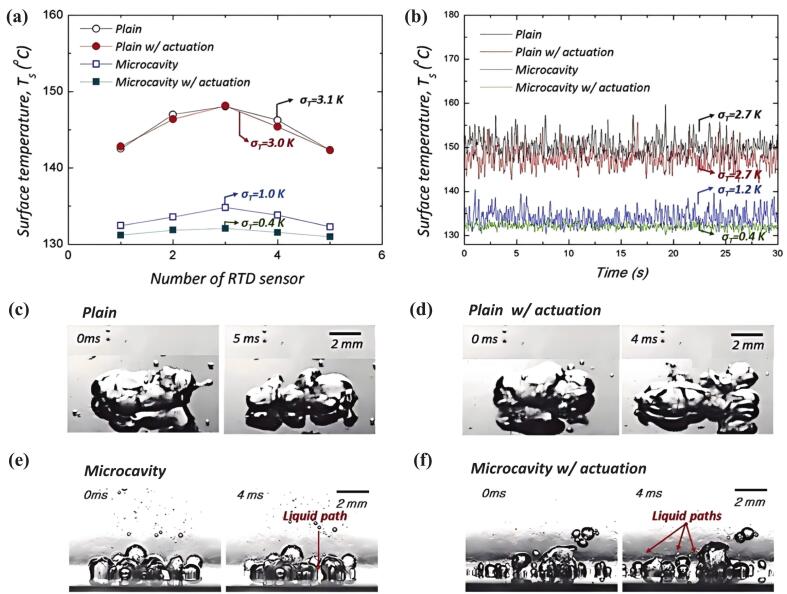
Thermal uniformity, stability, and bubble characteristics on plain and microcavity surfaces with and without ultrasonic actuation during the fully developed boiling region at heat flux of 2010 kW/m^2^ [155].

Microcavity structures enhanced heat transfer uniformity and stability by promoting orderly bubble departure via pinning effects and regular pitch. Ultrasonic actuation induced an acoustic field that acted on departed bubbles and increased the contact line instability between bubbles and microcavity structures, accelerating the dissipation of smaller bubbles. The combined use of microcavity structures and ultrasound resulted in smaller, faster, and more orderly bubble departure, which delayed coalescence and maintained liquid pathways, thereby improving CHF and thermal stability. Fig. 11 shows the thermal uniformity, stability, and bubble characteristics on plain and microcavity surfaces with and without ultrasonic actuation during the fully developed boiling region at heat flux of 2010 kW/m^2^ [155]. Combining rib surfaces with an ultrasonic field enhanced thermal performance more significantly than either single technology in pool boiling, due to the expanded heat transfer area and the combined cavitation and acoustic streaming effects. Rib arrangement was the most influential factor, followed by ultrasonic power and distance to the ultrasonic field [156]. The combination of cavity structures and ultrasound increased the acoustic wave penetration depth in the fluid, making it easier to induce acoustic streaming and thereby strengthening the heat transfer enhancement. Moreover, the efficiency of using ultrasound to enhance heat transfer was higher than that of using pump power [10]. Additionally, combining ultrasound with twisted tape effectively improved heat exchanger performance [124,157].

### 4.9 Others

**Fig. 12:**
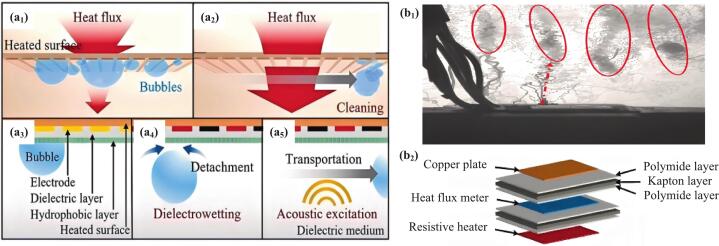
Integrated electric and acoustic actuation for heat transfer enhancement, a_3_-a_5_ are the sequential steps of bubble behavior by dielectrowetting and acoustic excitation [158]; Distribution of cavitation bubbles during the microgravity pool boiling heat transfer process (red circles indicate the regions of bubble cavitation at antinodes; red arrow indicates the trajectory of a bubble formed at the heater towards an antinode) [159]. (For interpretation of the references to colour in this figure legend, the reader is referred to the web version of this article.)

Researchers have also reported electro-acoustic coupled boiling heat transfer [158] and ultrasonic boiling heat transfer under microgravity condition [159]. In electro-acoustic coupled boiling, the synergistic regulation of bubble separation distances from the surface via dielectrowetting and detachment frequency through acoustic excitation enabled the simultaneous removal of multiple bubbles of various volumes for enhanced heat transfer [158], as shown in Fig. 12a. Acoustic actuation proved effective for enhancing heat transfer in microgravity when sufficiently high amplitudes were generated, especially near the actuator's nominal frequency. It reduced temperature, suppressed bubble formation, and controlled boil-off in propellant tanks. The heater substrate material influenced the acoustic field, affecting thermal behavior (Fig. 12b) [159]. In addition, the acoustic relocation of liquid based on nonlinear acoustic theory provided new insights for microgravity thermal management [160,161].

## 5 Concluding remarks

### 5.1 Summary of the existing research

(1) Ultrasound-enhanced heat transfer primarily relies on acoustic cavitation and acoustic streaming, with distinct mechanisms observed across different frequency ranges. Additional contributions arise from thermal effect (acoustic energy conversion to heat) and mid-to-high frequency-induced phenomena such as acoustic fountain and atomization at vapor–liquid interfaces. The dominance of these effects depends on a complex interplay of ultrasonic parameters, thermal–hydraulic conditions, and fluid properties.
(2) Studies reveal that ultrasound significantly improves heat transfer performance in different heat transfer systems. In single-phase convection, perpendicular ultrasonic standing waves enhance heat transfer, while parallel waves suppress it. For pool boiling, the most pronounced enhancement occurs at low heat fluxes, where ultrasound activates nucleation sites, reduces bubble detachment size, and delays dry-out. In forced convective boiling, low-frequency ultrasound excels at low *Re*, whereas high-frequency ultrasound relies on acoustic streaming, with diminishing effects at higher flow velocities. Heat exchangers benefit most from ultrasound at low flow rates, achieving heat transfer improvement with minimal friction loss.
(3) The influence of experimental parameters on heat transfer is significant. Low-frequency ultrasound enhances heat transfer through microjets and shock waves that disrupt thermal boundary layers, while high-frequency ultrasound generates macro- and micro-vortices. Increased ultrasonic power raises the HTC, but the enhancement ratio may decrease with power for some tube types. The effect diminishes with increasing flow rate, heat flux, and distance from or misalignment with the transducer. Nanofluids combined with ultrasound show enhanced HTC, particularly at higher concentrations and lower flow rates, though nanoparticle aggregation can attenuate ultrasonic waves. Surface structures like microcavities, ribs, and porous media synergize with ultrasound to promote bubble detachment, improve thermal stability, and increase CHF.
(4) Ultrasound regulates bubble dynamics, which is a key mechanism for enhancing heat transfer performance. The acoustic field induces capillary waves on bubble surfaces, generates primary and secondary Bjerknes forces, and promotes bubble oscillation, clustering, collapse, and detachment. These processes disrupt thermal boundary layers, induce micro-convection, and enhance fluid mixing. Factors such as bubble size, distance from the wall, liquid temperature, and surface roughness significantly influence the cavitation intensity and the resulting heat transfer enhancement.

### 5.2 Future research discussions on ultrasound-enhanced heat transfer

(1) Experimental studies on ultrasound-enhanced heat transfer currently face significant challenges due to the complex interplay of multiple parameters. Particularly under high-heat-flux conditions, pronounced attenuation of ultrasonic energy substantially diminishes the enhancement effectiveness. Furthermore, technical limitations persist in the dynamic monitoring of bubble behavior within microchannels, where conventional imaging techniques prove inadequate for accurately resolving vapor–liquid interface dynamics. To address these challenges, future research should employ advanced multimodal experimental approaches, integrating particle image velocimetry (PIV) with high-speed microimaging to systematically investigate boundary layer disturbance mechanisms. This methodology would enable the development of robust techniques for bubble characteristic parameter extraction and facilitate the construction of comprehensive heat transfer coefficient prediction models that account for coupled ultrasonic-thermal-flow interactions. Additionally, the implementation of adaptive ultrasonic control systems could dynamically optimize frequency and power parameters in real-time, thereby enhancing heat transfer stability across diverse operating conditions.
(2) While composite technologies combining ultrasound with nanofluids and porous structures demonstrate promising synergistic effects, their practical application is constrained by two critical challenges: insufficient energy conversion efficiency and contradictory findings regarding pressure drop characteristics. For instance, the incorporation of nanoparticles may significantly elevate flow resistance, while the acoustic streaming effect of high-frequency ultrasound exhibits notable attenuation under high flow rates. To overcome these limitations, future research should prioritize the development of intelligent material systems and advanced structural designs. This includes engineering gradient porous surfaces to optimize the balance between bubble dynamics and pressure drop characteristics, as well as integrating electro-magnetic-acoustic hybrid actuation systems for precise bubble manipulation. Equally important is the investigation of enhancement mechanisms under extreme operating conditions (including microgravity/hypergravity, high-pressure, and high-temperature environments), which could yield innovative thermal management solutions for aerospace applications.
(3) Numerical modeling approaches face significant limitations in multiscale coupling capabilities. The connection between microscopic bubble dynamics (described by the Rayleigh-Plesset equation) and macroscopic flow-heat transfer phenomena still relies heavily on empirical assumptions, while the conservation equations for phase-change processes remain incompletely solved. Moreover, the substantial scale discrepancy between simulated micrometer-sized bubbles and actual nanoscale nuclei introduces considerable prediction errors. To address these challenges, future modeling efforts should focus on developing advanced cross-scale coupling algorithms. Promising approaches include integrating molecular dynamics (MD) with CFD to achieve precise characterization of phase-change interfaces, combined with machine learning techniques (such as GMDH optimization models) to establish intelligent mappings between ultrasonic parameters and heat transfer performance. The implementation of high-performance computing architectures would further enhance simulation efficiency for complex operating conditions, enabling more accurate and comprehensive modeling of ultrasound-enhanced heat transfer systems.
(4) The interaction between ultrasonic disturbances and microchannel scale effect presents a significant challenge in maintaining flow stability. This complex phenomenon requires comprehensive investigation incorporating scale effect, multi-physics coupling, and working fluid properties to elucidate the fundamental relationship between interface dynamics and flow instability mechanisms. Future research should particularly focus on characterizing how vapor–liquid interface behavior influences wall temperature distribution and pressure drop fluctuation. Through systematic analysis of these interactions, the distinct flow instability modes and their transition conditions can be identified, ultimately enabling the development of robust instability criterion models.

## CRediT authorship contribution statement

**Chong Li:** Writing – review & editing, Writing – original draft, Validation, Supervision, Methodology, Investigation, Data curation, Conceptualization. **Zufen Luo:** Validation, Resources, Funding acquisition. **Yuchen Shao:** Validation. **Yuqi Qian:** Supervision. **Siliang Du:** Data curation, Conceptualization. **Quanquan Yang:** Resources. **Zhong Chen:** Supervision. **Hao Chen:** Investigation. **Yi Zha:** Visualization. **Xiande Fang:** Resources, Project administration.

## Declaration of competing interest

The authors declare that they have no known competing financial interests or personal relationships that could have appeared to influence the work reported in this paper.
